# Low Correlation Interference OFDM-NLFM Waveform Design for MIMO Radar Based on Alternating Optimization

**DOI:** 10.3390/s21227704

**Published:** 2021-11-19

**Authors:** Tianqu Liu, Jinping Sun, Qing Li, Zhimei Hao, Guohua Wang

**Affiliations:** 1 School of Electronics & Information Engineering, Beihang University, Beijing 100191, China; tqliu@buaa.edu.cn (T.L.); haozhimei@sina.com (Z.H.); 2 Department of Engineering, University of Cambridge, Cambridge CB2 1PZ, UK; ql289@cam.ac.uk; 3 Hertzwell Pte Ltd., Singapore 639798, Singapore; wghwood@hotmail.com

**Keywords:** OFDM, MIMO radar, NLFM, alternating optimization, particle swarm optimization (PSO), block coordinate descent (BCD), cross-correlation function, auto-correlation function sidelobe, sub-chirp rate

## Abstract

The OFDM chirp signal is suitable for MIMO radar applications due to its large time-bandwidth product, constant time-domain, and almost constant frequency-domain modulus. Particularly, by introducing the time-frequency structure of the non-linear frequency modulation (NLFM) signal into the design of an OFDM chirp waveform, a new OFDM-NLFM waveform with low peak auto-correlation sidelobe ratio (PASR) and peak cross-correlation ratio (PCCR) is obtained. IN-OFDM is the OFDM-NLFM waveform set currently with the lowest PASR and PCCR. Here we construct the optimization model of the OFDM-NLFM waveform set with the objective function being the maximum of the PASR and PCCR. Further, this paper proposes an OFDM-NLFM waveform set design algorithm inspired by alternating optimization. We implement the proposed algorithm by the alternate execution of two sub-algorithms. First, we keep both the sub-chirp sequence code matrix and sub-chirp rate plus and minus (PM) code matrix unchanged and use the particle swarm optimization (PSO) algorithm to obtain the optimal parameters of the NLFM signal’s time-frequency structure (NLFM parameters). Next, we keep current optimal NLFM parameters unchanged, and optimize the sub-chirp sequence code matrix and sub-chirp rate PM code matrix using the block coordinate descent (BCD) algorithm. The above two sub-algorithms are alternately executed until the objective function converges to the optimal solution. The results show that the PASR and PCCR of the obtained OFDM-NLFM waveform set are about 5 dB lower than that of the IN-OFDM.

## 1. Introduction

**Figure 1:**
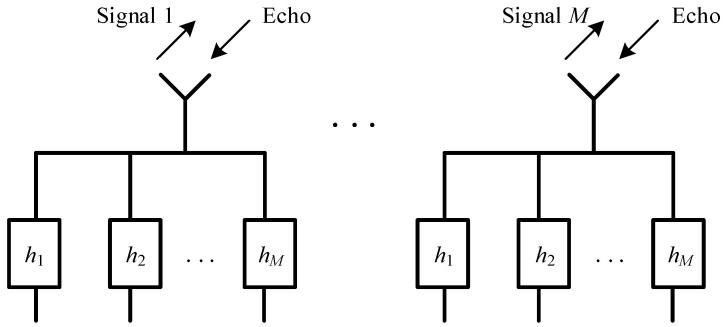
A monostatic MIMO radar with *M* antennas for transmitting and receiving.

Multiple-input multiple-output (MIMO) radar uses waveform diversity techniques to improve power efficiency, clutter suppression ability, and other performances. A monostatic MIMO radar with *M* transmitting/receiving antennas is shown in Figure 1. The *M* signals transmitted by the *M* antennas are different. The echo of all transmitted signals are collected by each receiving antenna. MIMO diversity gain is achieved by an ideal separation of the echo. The filter *h_m_*, *m* = 1, 2, …, *M* is matched to the *m*-th transmit signal. The echo from the other *M*-1 transmitted signals causes correlation interference at the output of *h_m_*. A MIMO waveform set is orthogonal when the cross-correlation function between any pair of *M* transmit signals is zero. Although orthogonality is not achievable within a limited time-bandwidth product, a low cross-correlation is achievable. Furthermore, the sidelobe of the auto-correlation of the waveform set should be lowered to improve pulse compression performance. The research of the MIMO radar waveform design focuses on reducing the peak of auto-correlation function sidelobe and cross-correlation function within a limited time-bandwidth product [1]. Orthogonal frequency division multiplexing (OFDM) signal is suitable for application in MIMO radar systems because of its high range resolution, high Doppler resolution, and high design freedom [2,3,4,5,6,7].

There has been much research on the OFDM waveform for single-input single-output (SISO) radar systems. Initially, OFDM waveforms were used in multi-carrier FMCW radar systems [8]. Subsequent studies of the OFDM waveform have adopted this modulation strategy of sub-carriers in their framework. However, most existing radar OFDM waveforms have a non-constant time-domain modulus. As the power amplifiers of real radar systems are generally non-linear, it is critical to reduce the peak-to-mean envelope power ratio (PMEPR) and peak-to-average power ratio (PAPR) of OFDM waveforms. At the same time, it is also important to optimize the performance of the auto-correlation function and ambiguity function [9,10,11]. To solve this problem, practical radar systems can use power amplifiers with higher dynamic range and linearity, but the trade-offs are higher system cost and complexity. Several researchers have evaluated the impact of directly limiting the amplitude of the OFDM waveform, and showed that the magnitude of noise and interference increases [12]. Besides, the direct design of the constant envelope OFDM waveform is another way. R. Mohseni et al. [13] and Wen-Qin Wang et al. [14] have proposed several constant envelope OFDM waveforms with good correlation performance and flexible parameter design.

In recent years, with the development of MIMO radar, there has been an increasing number of studies on designing OFDM waveform sets for MIMO radar systems. According to the different sub-carriers, the MIMO radar OFDM waveform sets are divided into two categories: LFM-OFDM [15,16,17,18,19,20] and PC-OFDM [21,22,23,24,25]. The sub-carrier of the LFM-OFDM waveform set is a chirp signal, and thus, the LFM-OFDM waveform set has better Doppler tolerance. The PC-OFDM waveform set consists of multi-carrier phase-coded signals. Comprehensive and in-depth studies on single-carrier phase-coded waveform set for MIMO radar can be found in [26,27,28,29], and not many problems are left in the multi-carrier case. LFM-OFDM waveform sets have better prospects in MIMO-SAR systems due to the advantages of chirp signals [30,31,32]. Wen-Qin Wang [33] has designed a constant envelope MIMO radar LFM-OFDM waveform set, termed OFDM chirp, modulated by random sub-chirp codes. OFDM chirp waveform set has relatively high cross-correlation function peaks [34]. If its cross-correlation peaks are reduced to a certain extent, the OFDM chirp waveform can be applied to not only MIMO synthetic aperture radar (SAR) systems [35,36], but also other MIMO radar systems [37]. Compared with LFM signals, non-linear frequency modulation (NLFM) signals have a lower auto-correlation sidelobe and a larger degree of freedom. Based on the time-frequency structure of NLFM signals, a possible approach to suppress the correlation peaks of the OFDM chirp waveform set is to use different sub-chirp rates, e.g., Gao et al. [38] use the time-frequency structure of the NLFM signals to construct the sub-chirps of the OFDM chirp waveform set with lower cross-correlation peaks. The obtained new waveform set is referred to as the OFDM-NLFM waveform set in this paper. Since then, there have been several studies on the OFDM-NLFM waveform set [39,40]. Currently, the IN-OFDM designed by Xiang Lan et al. [41] is the OFDM-NLFM waveform set with the lowest peak of auto-correlation sidelobe ratio (PASR) and cross-correlation ratio (PCCR).

This paper develops the signal model and the optimization model of the OFDM-NLFM waveform set. The time-frequency structure of the OFDM-NLFM waveform set is constructed by sub-chirp signals with different time-width, center frequency, and chirp rates. After determining the number of transmitted signals of the OFDM-NLFM waveform set, the number of sub-bands, and other system parameters, an OFDM-NLFM waveform set is generated by the following steps. The first step is to initialize the NLFM signal’s time-frequency structure (NLFM parameters). Then, the time-frequency structure of the NLFM signal is divided into several sub-bands, or sub-pulses. Next, we replace the nonlinear frequency modulation curve in each sub-band with a linear frequency modulation curve and generate the piecewise chirp signal to approximate the NLFM signal. Finally, the sequence order of sub-chirp signals is permutated according to the sub-chirp sequence code matrix, and the sign of chirp rates is changed according to the sub-chirp rate plus and minus (PM) code matrix. Especially, each row of the sub-chirp sequence code matrix is a permutation that determines the order of the sub-chirp sequence for each transmit signal of the OFDM-NLFM waveform set, and each element of the sub-chirp rate PM code matrix is a 1-bit binary number that determines whether the sub-chirp signal is an up-chirp signal or a down-chirp signal. This paper defines the objective function as the sum of the cross-correlation functions considering that the correlation interferences from the other transmit signals are additive.

To design the OFDM-NLFM waveform set with the lowest possible PASR and PCCR, this paper proposes an OFDM-NLFM waveform set design algorithm based on alternating optimization. There are both discrete code matrices and continuous NLFM parameters of the OFDM-NLFM waveform set. The expression between the objective function and the waveform parameters consists of time-frequency structure transformation and correlation function calculation. Hence, the OFDM-NLFM waveform set design problem is a high-dimensional optimization problem with a complex objective function and complicated optimization variables. Dimensionality reduction and decomposition are efficient solutions to a high-dimensional optimization problem with fast execution [42,43,44]. The method proposed in this paper adopts the idea of the alternating direction multiplier method (ADMM) [45], which is also known as alternating optimization. The parameters of the OFDM-NLFM waveform set to be optimized include continuous and discrete variables. For continuous variables, using the particle swarm optimization (PSO) algorithm [46] to find the minimum value of the complex objective function is simple and efficient. For discrete variables, it is difficult to find the optimal solution analytically since the sub-chirp sequence code matrix and the sub-chirp rate PM code matrix are unconstrained. The coordinate descent (CD) algorithm [47] is efficient to solve the unconstrained optimization problem. Inspired by the CD algorithm, a new block coordinate descent (BCD) algorithm is proposed to find the optimal solution of the sub-chirp sequence code matrix and the sub-chirp rate PM code matrix. In this method, the optimization parameter matrices are divided into blocks according to different transmit signals that they determine. In short, the OFDM-NLFM waveform set design algorithm based on alternating optimization is composed of two sub-algorithms; the PSO algorithm optimizes the continuous NLFM parameters, and the BCD algorithm optimizes the sub-chirp sequence code matrix and the sub-chirp rate PM code matrix. After either of the two sub-algorithms has been executed, the optimal result is passed to the other sub-algorithm, ensuring that the objective function decreases monotonically until it converges.

This paper develops the signal model and parameter optimization model of the OFDM-NLFM waveform set. A novel OFDM-NLFM waveform set design algorithm based on alternating optimization is proposed. With the same system parameters as the current optimal IN-OFDM waveform, the PASR and PCCR of the obtained waveform set are about 5 dB lower than that of the IN-OFDM. The rest of this paper is organized as follows. Section 2 introduces the signal model of the OFDM-NLFM waveform set and establishes the mathematical model of MIMO radar OFDM-NLFM waveform set optimization design problem. Section 3 presents the details of the OFDM-NLFM waveform set design algorithm based on alternating optimization. Section 4 performs numerical simulations under different system parameters. The results are compared with the current optimal OFDM-NLFM waveform set, IN-OFDM. The influences of different parameters on the algorithm design results are analyzed. Section 5 is the conclusion.

## 2. Signal Model and Optimization Model

### 2.1. OFDM-NLFM Waveform Set Signal Model

Consider a monostatic MIMO radar with *M* antennas for transmitting and receiving. An OFDM-NLFM waveform set can be generated as follow. (1) construct the time-frequency structure of the piecewise chirp signal that approximates the origin NLFM signal. Note that the sub-chirp signals of the piecewise chirp signal have different sub-chirp rates and bandwidths. (2) minimize the cross-correlation function by adjusting the sub-chirps order in the time-frequency structure of the piecewise chirp signal. Therefore, the parameters of the OFDM-NLFM waveform set consist of two parts; for each transmit signal, one part is NLFM parameters that control the time-frequency structure, and another part is the sub-chirp sequence code matrix and the sub-chirp rate PM code matrix that controls the order of sub-chirp sequence and the sub-chirp rates, respectively.

**Figure 2:**
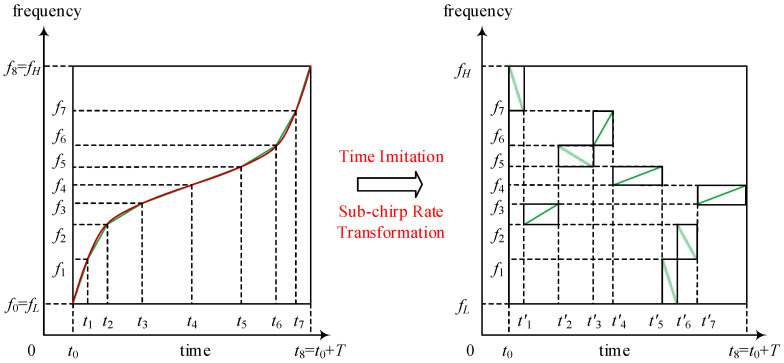
The time-frequency structure of one of *M* transmit signals of the OFDM-NLFM waveform set.

Figure 2 is the time-frequency structure of one of the *M* transmit signals of the OFDM-NLFM waveform set. Each signal of the OFDM-NLFM waveform set is generated by a time-imitating and sub-chirp rate transforming of a piecewise chirp signal. In this figure, the number of sub-pulses and sub-bands is set to *N =* 8. *T* represents pulse duration. *f_L_* and *f_H_* represent the highest and lowest signal frequency, respectively. The red curve on the left represents the time-frequency structure of the original NLFM signal, and the green broken line that approximates the red curve represents the time-frequency structure of the corresponding piecewise chirp signal. The values of $t_{0},t_{1},\ldots ,t_{8}$ and $f_{0},f_{1},\ldots ,f_{8}$ in Figure 2 determine the segmentation points of the time-frequency structure curve. Subsequently, the sequence order of the sub-chirp signals is permutated, and the signs of the sub-chirp rates are adjusted. It is noticed that permutating the sequence order of the sub-chirp signals can be completed in both the time and frequency domain.

The first step to constructing the signal shown in Figure 2 is to generate an NLFM signal and a piecewise chirp signal. The time-frequency structure of the NLFM signal depends on its signal model and parameters. Based on the time-frequency structure of the chirp signal, we generate the time-frequency curve *f*(*t*) of the NLFM signal by adding several sine function items whose amplitude is controlled by parameters [48]. The time-frequency structure curve established in this way is defined as

(1) $$\left\{ \begin{array}{l} y(x)=\frac{B}{T}x+a\cdot \frac{B}{\pi }\sin(\frac{2\pi }{T}x)+b\cdot \frac{B}{\pi }\sin(\frac{4\pi }{T}x)\\ f(t)=y(t-t_{0}-\frac{T}{2}) \end{array}\right.,$$

where *a* and *b* are adjustable parameters. *t* and *f* represent the time and instantaneous frequency, respectively, $t_{0}\leq t\leq t_{0}+T$ and $f_{0}\leq f\leq f_{0}+B$. The time-frequency curve is limited to a rectangle with a bandwidth of $B=f_{H}-f_{L}$ and time width *T*. One way to determine the segmentation points of the time-frequency curve is to set time domain segmentation points, which can be expressed as

(2) $$f_{n}=f(t_{n}),\;n=0,1,2,\ldots ,N.$$

Another way is to set frequency segmentation points at first. The segmentation points of the time-frequency curve are determined according to the reverse function of *f*(*t*) in Equation (1), expressed as

(3) $$t_{n}=f^{-1}(f_{n}),\;n=0,1,2,\ldots ,N,$$

where *f*^−1^ represents the reverse function of *f*(*t*). Numerical methods can be adopted when the reverse function is difficult to solve. Equations (2) and (3) show that any two of the three, which are the NLFM parameters, time-domain segmentation points, and frequency-domain segmentation points, determine the time-frequency structure of the piecewise chirp signal. According to the obtained time-frequency structure, the time width, sub-chirp rate, and the carrier frequency of each sub-chirp signal can be determined. The time width is

(4) $$T_{n}=t_{n}-t_{n-1},\;n=1,2,\ldots ,N.$$

The sub-chirp rate is

(5) $$k_{n}=\frac{B_{n}}{T_{n}}=\frac{f_{n}-f_{n-1}}{t_{n}-t_{n-1}},\;n=1,2,\ldots ,N.$$

The center frequency of each sub-band is

(6) $$f_{cn}=\frac{1}{2}\left(f_{n}+f_{n-1}\right),\;n=1,2,\ldots ,N.$$

The sign of the sub-chirp rate is a vector defined as

(7) $$\alpha =\left[\alpha_{1},\ldots ,\alpha_{n},\ldots ,\alpha_{N}\right],\;\alpha_{n}\in \left\{1,-1\right\}.$$

According to Equations (4)–(7), the complex expression of each sub-chirp signal is

(8) $$s_{n}(t)=\exp(j\pi \alpha_{n}k_{n}t^{2})\cdot \exp(j2\pi f_{cn}t),\;t\in \left[-\frac{T_{n}}{2},\frac{T_{n}}{2}\right).$$

The sub-chirp signals $s_{n}(t)$ of a piecewise chirp signal are shown in Equation (8). As shown in Figure 2, the time-imitation of the piecewise chirp signal is required to obtain each of the *M* transmit signals of the OFDM-NLFM waveform set. The sub-chirp sequence code that determines the order of the time-imitation is defined as

(9) $$\beta =\left[\beta_{1},\beta_{2},\ldots ,\beta_{N}\right],$$

where $\beta_{1},\beta_{2},\ldots ,\beta_{n},\ldots ,\beta_{N}$ is a permutation of 1,2,…,N. Note that the *n*-th sub-chirp signal after the time-imitation equals the $\beta_{n}$-th sub-chirp signal before the time-imitation. For the convenience of derivation, let $t_{0}=0$ in the following. The expression of one of *M* transmit signals of the OFDM-NLFM waveform set is

(10) $$s(t)=\sum_{n=1}^{N}rect(\frac{t+\frac{1}{2}T_{\beta_{n}}-\sum_{i=1}^{n}T_{\beta_{i}}}{T_{\beta_{n}}})s_{\beta_{n}}(t+\frac{1}{2}T_{\beta_{n}}-\sum_{i=1}^{n}T_{\beta_{i}}),$$

where *rect*(*x*) is the rectangular window function

(11) $$rect(x)=\left\{ \begin{array}{ll} 1 & \left|x\right|\leq 0.5\\ 0 & \left|x\right|>0.5 \end{array}\right..$$

For the OFDM-NLFM waveform set with *M* transmit signals, we have the following waveform parameters:

(1) A total of *M* groups time-frequency structure curve parameters of the NLFM signal (NLFM parameters) can be defined as
  (12) $$\left[\left(a_{1},b_{1}\right),\left(a_{2},b_{2}\right),\ldots ,\left(a_{M},b_{M}\right)\right].$$
(2) Segmentation points parameters in the time domain or frequency domain. The segmentation points and the NLFM parameters in Equation (12) determine the sub-chirp rate, center frequency, time width, and bandwidth of the sub-chirp signal in the sub-band. For the *m*-th transmit signal, the time domain segmentation points are $t_{1}^{m},t_{2}^{m},\ldots ,t_{n}^{m},\ldots ,t_{N}^{m}$ *M* groups segmentation parameters can be defined as
  (13) $$\left[\begin{array}{cccc} t_{1}^{1} & t_{2}^{1} & \cdots & t_{N}^{1}\\ t_{1}^{2} & t_{2}^{2} & \cdots & t_{N}^{2}\\ \vdots & \vdots & \ddots & \vdots \\ t_{1}^{M} & t_{2}^{M} & \cdots & t_{N}^{M} \end{array}\right].$$
(3) The sub-chirp sequence code matrix and the sub-chirp rate PM code matrix. Both matrices are composed of *M* row vectors. The meaning of every element of the matrix can be seen in Equations (7) and (9). The two matrices can be defined in Equation (14), where $\alpha_{n}^{m}\in \left\{1,-1\right\},m=1,2,\ldots ,M$, $\beta_{1}^{m},\beta_{2}^{m},\ldots ,\beta_{n}^{m},\ldots ,\beta_{N}^{m}$ is a permutation of 1,2,…,N.
  (14) $$C_{1}=\left[\begin{array}{cccc} \alpha_{1}^{1} & \alpha_{2}^{1} & \cdots & \alpha_{N}^{1}\\ \alpha_{1}^{2} & \alpha_{2}^{2} & \cdots & \alpha_{N}^{2}\\ \vdots & \vdots & \ddots & \vdots \\ \alpha_{1}^{M} & \alpha_{2}^{M} & \cdots & \alpha_{N}^{M} \end{array}\right],C_{2}=\left[\begin{array}{cccc} \beta_{1}^{1} & \beta_{2}^{1} & \cdots & \beta_{N}^{1}\\ \beta_{1}^{2} & \beta_{2}^{2} & \cdots & \beta_{N}^{2}\\ \vdots & \vdots & \ddots & \vdots \\ \beta_{1}^{M} & \beta_{2}^{M} & \cdots & \beta_{N}^{M} \end{array}\right].$$

According to the above three groups’ parameters, the expression of the *m*-th transmit signal of the MIMO radar OFDM-NLFM waveform set is

(15) $$s^{m}(t)=\left\{ \begin{array}{ccc} \sum_{n=1}^{N}rect\left(\frac{t+\frac{1}{2}T_{\beta_{n}^{m}}^{m}-\sum_{i=1}^{n}T_{\beta_{i}^{m}}^{m}}{T_{\beta_{n}^{m}}^{m}}\right)s_{\beta_{n}^{m}}^{m}(t+\frac{1}{2}T_{\beta_{n}^{m}}^{m}-\sum_{i=1}^{n}T_{\beta_{i}^{m}}^{m}) & , & 0\leq t\leq T\\ 0 & , & \mathrm{others} \end{array}\right.,$$

where m=1,2,…,M, and each sub-chirp signal is defined as

(16) $$s_{\beta }^{m}(t)=\exp(j\pi \alpha_{\beta }^{m}k_{\beta }^{m}t^{2})\cdot \exp(j2\pi f_{c\beta }^{m}t),\;t\in \left[-\frac{T_{\beta }^{m}}{2},\frac{T_{\beta }^{m}}{2}\right),\beta =\beta_{n}^{m},$$

where $k_{\beta }^{m},f_{c\beta }^{m},T_{\beta }^{m}$ can be calculated according to Equations (3)–(6).

### 2.2. MIMO Radar Waveform Set Performance Evaluation

For a MIMO radar waveform set with *M* transmit signals, the aperiodic auto- and cross-correlation function for each pair of signals are

(17) $$R_{i}(t)=\int_{-T}^{T}s_{i}(s)s_{i}^{*}(s-t)ds,\;i\in \left\{1,2,\ldots ,M\right\},$$

(18) $$R_{ij}(t)=\int_{-T}^{T}s_{i}(s)s_{j}^{*}(s-t)ds,\;i\neq j\;\mathrm{and}\;i,j\in \left\{1,2,\ldots ,M\right\},$$

where $R_{i}(t)$ represents auto-correlation function and $R_{ij}(t)$ represents cross-correlation function. In a real MIMO radar system, the echo of all transmit signals enters into each matched filter. Therefore, the waveform set performance should be a cross-correlation function between the sum signal of the other transmit signals and itself. Here we define the correlation functions for MIMO radar as follows.

(19) $$AR_{i}(t)=\int_{-T}^{T}s^{i}(s)s^{i*}(s-t)ds,\;i\in \left\{1,2,\ldots ,M\right\},$$

(20) $$CR_{i}(t)=\int_{-T}^{T}s^{i}(s)\left[\sum_{1\leq j\leq M,j\neq i}s^{j*}(s-t)\right]ds,\;i\in \left\{1,2,\ldots ,M\right\},$$

where $AR_{i}(t)$ and $CR_{i}(t)$ represent auto- and cross-correlation function of the *i*-th transmit signal of the waveform set. Ideal orthogonality means $CR_{i}(t)=0$ for any *t* and *i* in Equation (20), which is not achievable. In order to optimize the orthogonality and pulse compression performance of the OFDM-NLFM waveform set, the following indicators are optimized by the proposed OFDM-NLFM waveform set design algorithm.

(21) $$\mathrm{PASR}=\underset{1\leq i\leq M}{\max}10\log_{10}\left(\underset{\begin{array}{c} t\\ \left|t\right|\geq w \end{array}}{\max}{\left|\frac{AR_{i}(t)}{AR_{i}(0)}\right|}^{2}\right)\;\mathrm{dB},$$

(22) $$\mathrm{PCCR}=\underset{1\leq i\leq M}{\max}10\log_{10}\left(\underset{t}{\max}{\left|\frac{CR_{i}(t)}{AR_{i}(0)}\right|}^{2}\right)\;\mathrm{dB}.$$

In Equations (21) and (22), $AR_{i}(0)$ is the peak value of the auto-correlation function, and *w* is the width of the auto-correlation mainlobe. PASR is the peak auto-correlation sidelobe ratio, and PCCR is the peak cross-correlation ratio. The waveform set performs better when its values are lower.

### 2.3. OFDM-NLFM Waveform Set Optimization Model

Based on the signal model of the OFDM-NLFM waveform set and MIMO radar correlation function performance indicators, this section establishes the optimization model for the OFDM-NLFM waveform set. The solution space of this optimization problem consists of the OFDM-NLFM waveform set parameters, including the NLFM parameters, the sub-chirp sequence code matrix, and the sub-chirp rate PM code matrix. Here we do not optimize the segmentation parameters. One reason is that the dimension of the optimization variable will be much higher if the segmentation parameters are included. Another reason is that it is tricky to optimize it using the existing continuous parameter optimization method, as the mapping between time-frequency segmentation parameters and the objective function is very complicated. Therefore, the optimization model established in this paper does not consider the time-frequency segmentation parameters.

The solution space and optimization variables of the optimization problem are modeled as

(23) $$\left\{ \begin{array}{l} X=(X_{1},X_{2},X_{3})\in \Omega \\ X_{1}=\left[\begin{array}{cccc} \begin{array}{l} a_{1}\\ b_{1} \end{array} & \begin{array}{l} a_{2}\\ b_{2} \end{array} & \begin{array}{l} \cdots \\ \cdots \end{array} & \begin{array}{l} a_{M}\\ b_{M} \end{array} \end{array}\right],X_{2}=\left[\begin{array}{cccc} \alpha_{1}^{1} & \alpha_{2}^{1} & \cdots & \alpha_{N}^{1}\\ \alpha_{1}^{2} & \alpha_{2}^{2} & \cdots & \alpha_{N}^{2}\\ \vdots & \vdots & \ddots & \vdots \\ \alpha_{1}^{M} & \alpha_{2}^{M} & \cdots & \alpha_{N}^{M} \end{array}\right],X_{3}=\left[\begin{array}{cccc} \beta_{1}^{1} & \beta_{2}^{1} & \cdots & \beta_{N}^{1}\\ \beta_{1}^{2} & \beta_{2}^{2} & \cdots & \beta_{N}^{2}\\ \vdots & \vdots & \ddots & \vdots \\ \beta_{1}^{M} & \beta_{2}^{M} & \cdots & \beta_{N}^{M} \end{array}\right] \end{array}\right..$$

The solution space Ω is composed of three independent sub-spaces, and its optimization variable *X* is divided into three parts corresponding to the OFDM-NLFM waveform set parameters shown in Equations (12) and (14).

According to the definition of the solution space and the OFDM-NLFM waveform set, the mapping from the solution space to the objective function domain is described as

(24) $$\left\{ \begin{array}{l} ℱ\left(X\right)=F_{2}\left(s\right)=F_{2}\left(F_{1}\left(X\right)\right)\\ F_{2}\left(s\right)=\max\left\{\mathrm{PASR}\left(s\right),\mathrm{PCCR}\left(s\right)\right\}\\ s=s(t)=\left[s^{1}(t),s^{2}(t),\ldots ,s^{M}(t)\right]\in S \end{array}\right.,$$

where *F*_2_ represents the mapping from the waveform set *s*(*t*) to the objective function. The two performance indicators, PASR and PCCR, are actually functions of *s*(*t*). *F*_1_ represents the mapping from the solution space Ω to the signal space *S*. According to the definition of the solution space and objective function, the OFDM-NLFM waveform set optimization problem of MIMO radar is modeled as

(25) $$\begin{array}{l} \underset{X}{\min}ℱ\left(X\right)=F_{2}\left(F_{1}\left(X\right)\right)=F_{2}\left({ϒ}_{2}\left({ϒ}_{1}\left({ϒ}_{0}\left(X_{1},t_{1},t_{2},\ldots ,t_{N}\right),X_{2}\right),X_{3}\right)\right)\\ \;s.t.\;X=(X_{1},X_{2},X_{3})\in \Omega \end{array},$$

where ℱ(X) is the objective function. Ω is the solution space. *X* is an optimization variable satisfying the constraint conditions in Equation (23). ${ϒ}_{0}$ represents the mapping from the solution space Ω to the space of piecewise chirp signal. $t_{1},t_{2},\ldots ,t_{n},\ldots ,t_{N}$ are the time-domain segmentation parameters. ${ϒ}_{1}$ and ${ϒ}_{2}$ are sub-chirp rate transformation and time-imitation for the piecewise chirp signal obtained by ${ϒ}_{0}$, respectively. *X*_2_ controls the sign of the sub-chirp rate in each sub-bands. *X*_3_ controls the sequence order of each sub-chirp signal. In short, the optimized objective function value ℱ(X) is obtained by first mapping every variable *X* in the solution space Ω to the waveform set space *S*, and then mapping the obtained OFDM-NLFM waveform set to the objective function domain.

## 3. OFDM-NLFM Waveform Set Design Algorithm Based on Alternating Optimization

In the above-mentioned MIMO radar OFDM-NLFM waveform set optimization problem, the solution space is of high dimension, and its objective function is complex. This paper proposes an OFDM-NLFM waveform set design algorithm based on alternating optimization. Especially, we divide the solution space into sub-spaces according to its characteristics, and the optimization problem in sub-spaces is viable to solve.

For an OFDM-NLFM waveform set with *M* transmit signals, each of which has *N* sub-chirp signals, the optimization variable *X* includes three parts. *X*_1_ is NLFM parameters consisting of 2*M* continuous variables. *X*_2_ is a matrix consisting of *MN* binary bits, with 2*^MN^* different values. *X*_3_ consists of *M* permutations, with a total of (*N*!)*^M^* different values. Thus the dimension of the solution space Ω is very high, and the structure of the optimization variable is complex containing continuous, discrete variables and permutations. To map from the solution space to the objective function domain, we first map the solution space to the waveform, and then calculate the performance indicators of the MIMO waveform set. A difficulty is that the simple auto- and cross-correlation functions are not elementary functions, but instead the functionals of the waveform set. To conclude, the objective function does not possess good properties such as continuity and derivability. The OFDM-NLFM waveform set optimization problem defined in Equation (25) is an NP-hard constrained optimization problem with mixed discrete and continuous optimization variables. Its objective function is nonlinear, nonconvex, and non-differentiable. In this case, the common linear programming, continuous optimization, and convex optimization methods fail, and the other optimization design algorithms for the MIMO radar waveform set cannot apply to the OFDM-NLFM waveform set as they are limited to its specific waveform set.

As the objective function is difficult to be simplified, the most direct approach is to separate discrete variables from continuous variables. Alternating optimization is a computational framework to solve high-dimensional optimization problems [45]. Alternating optimization decomposes the large global problem into smaller and easier-solved sub-problems by coordinate decomposition and obtains the solution of the global problem by combining the solutions of the sub-problems. The OFDM-NLFM waveform set design algorithm proposed by this paper is based on the idea of alternating optimization. If we keep the continuous variables unchanged and optimize the discrete variables, the problem is a combinatorial optimization problem. Instead, if we keep the discrete variables unchanged, the problem is a continuous optimization problem. This paper selects appropriate optimization sub-algorithms to solve each of the two problems. After one sub-algorithm has been executed, the current optimal solution is passed to the input of the other sub-algorithm.

The optimization of the discrete variable of the OFDM-NLFM waveform set is an unconstrained combinatorial optimization problem. Since the dimension of the discrete variable is very large, it is not feasible to find the global optimal solution accurately. Thus, the CD algorithm can be used to solve the unconstrained optimization problem by conducting approximate minimization along the coordinate direction or in the coordinate hyperplane [47]. This paper optimizes the sub-chirp sequence code matrix and the sub-chirp rate PM code matrix based on the idea of the CD algorithm. The CD algorithm is fast in execution, but its optimization result could be unstable. To balance the efficiency and stability of the CD algorithm, the parameter matrices are divided into blocks according to the different transmit signals that they determine. Each row of the sub-chirp sequence code matrix and the sub-chirp rate PM code matrix corresponds to each transmit signal of the OFDM-NLFM waveform set. The BCD algorithm optimizes one row of the matrix with the other rows remaining unchanged. In this way, it can greatly reduce the computational complexity of the algorithm when optimizing the high dimensional matrices.

The intelligent optimization algorithm is a simple and direct solution for the continuous parameters of the OFDM-NLFM waveform set, considering that the objective function is very complex and difficult to be simplified and transformed. The PSO algorithm is a random search algorithm originally inspired by the foraging behavior of birds [46]. It is found to be suitable for dealing with high-dimensional continuous variable optimization problems with the complex objective function. Hence, in this paper, the PSO algorithm is used to optimize NLFM parameters.

**Figure 3:**
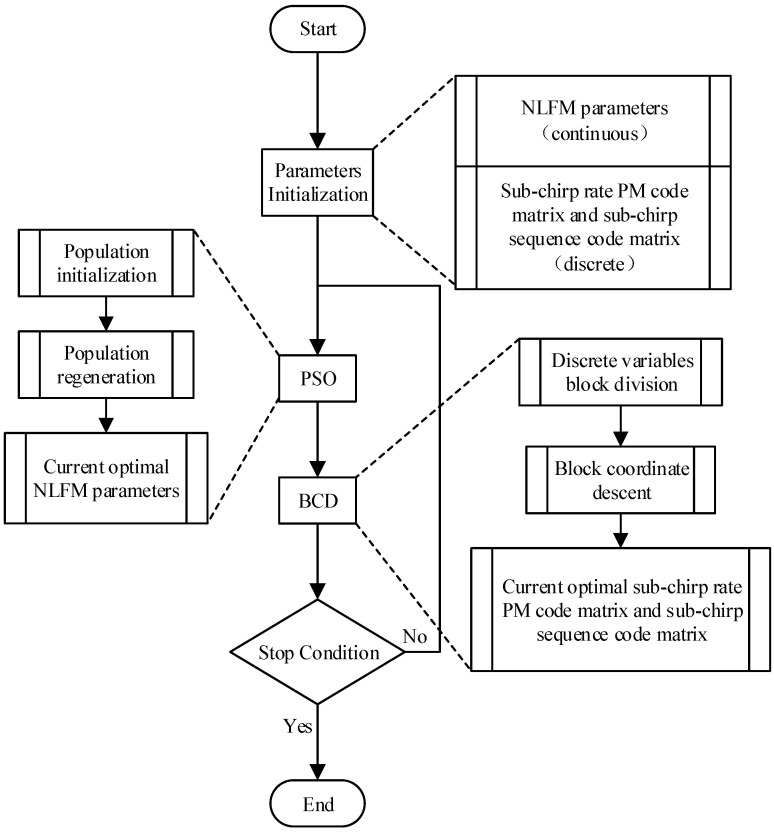
Block diagram of proposed OFDM-NLFM waveform set design algorithm.

The overall block diagram of the proposed OFDM-NLFM waveform set design algorithm is shown in Figure 3, and the specific steps of the algorithm are as follows.

**Step 1:** Select the initial NLFM parameters and construct *M* time-frequency curves according to Equation (1). The sub-chirp rate PM code matrix with *MN* elements is generated by random binary numbers. The sub-chirp sequence code matrix is generated by *M* random permutations. *M* is the number of transmit signals, and *N* is the number of the sub-chirp signals of each transmit signal.

**Step 2:** After the parameter initialization step, the time domain segmentation parameters in Equation (13) should be set properly.

**Step 3:** After the initial waveform has been obtained, keep the sub-chirp rate PM code matrix and sub-chirp sequence code matrix unchanged. Optimize NLFM parameters using the PSO algorithm and obtain the current optimal NLFM parameters. Before executing, the PSO algorithm, population size, the maximum number of iterations, and other parameters should be set properly.

**Step 4:** Keep the current optimal NLFM parameters obtained in step 3 unchanged, sub-chirp rate PM code matrix and sub-chirp sequence code matrix are optimized based on the BCD algorithm. Firstly, the matrix to be optimized is divided into several small blocks. Secondly, each block is optimized in turns with other blocks unchanged. Finally, the current optimal sub-chirp rate PM code matrix and sub-chirp sequence code matrix are obtained.

**Step 5:** If the difference between the objective function values before and after step 3 and step 4 is lower than the threshold, the optimization algorithm converges. Output the current optimal NLFM parameters, sub-chirp rate PM code matrix, and sub-chirp sequence code matrix. Otherwise, jump to step 3 and continue.

The specific implementation of the above steps is described in the following. Section 3.1 introduces the initialization of OFDM-NLFM waveform set parameters. Section 3.2 introduces the optimization of NLFM parameters based on the PSO algorithm. Section 3.3 introduces the optimization of sub-chirp rate PM code matrix and sub-chirp sequence code matrix based on the BCD algorithm.

### 3.1. OFDM-NLFM Waveform Set Parameter Initialization

The principle of the parameter initialization for the OFDM-NLFM waveform set is to make the peak of the auto-correlation function sidelobe low. The NLFM parameters in Equation (12) affect the peak of the auto-correlation function sidelobe, measured by the PASR in Equation (21). In order to help select the appropriate initial values of the NLFM parameters, this section traverses the parameter value in a certain range to test its impact on the PASR. The PASR is also affected by the sub-chirp rate PM code matrix and sub-chirp sequence code matrix. Therefore, in this section, the PASR of *M* transmit signals is tested under several groups of random initialed sub-chirp rate PM code matrices and sub-chirp sequence code matrices. For each group of the matrices, the test goes through all the values of NLFM parameters with a∈(−0.9,0.9),b∈(−1,1). 

**Figure 4:**
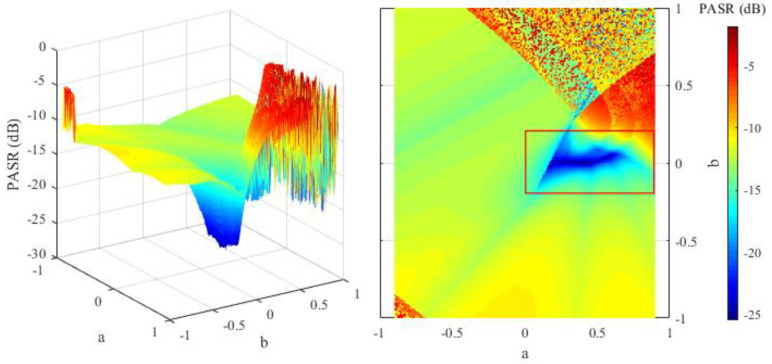
PASR values of different NLFM parameters under group 1 of sub-chirp rate PM code matrix and sub-chirp sequence code matrix.

**Figure 5:**
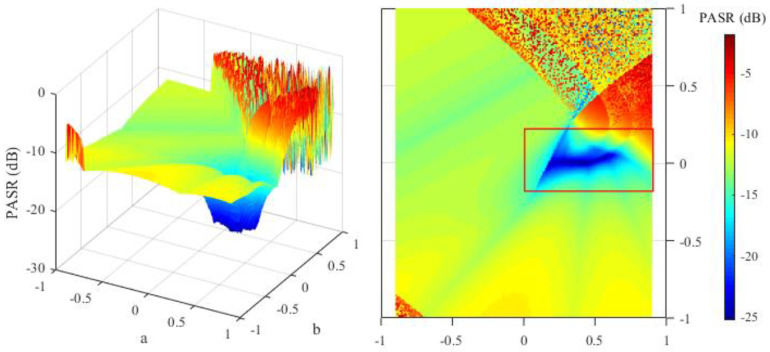
PASR values of different NLFM parameters under group 2 of sub-chirp rate PM code matrix and sub-chirp sequence code matrix.

Set *M* = 1 in Equations (12) and (21) because a bigger *M* is not necessary when PCCR is not analyzed. The PASR under two different groups of sub-chirp rate PM code matrices and sub-chirp sequence code matrices are shown in Figure 4 and Figure 5. The results show that the PASR is lower when the NLFM parameters are in the interval of a∈(0,0.9),b∈(−0.2,0.2). Thus, the initial values of NLFM parameters should be selected from the above interval. It can be seen from Figure 4 and Figure 5 that different sub-chirp rate PM code matrix and sub-chirp sequence code matrix have little impact on PASR. Therefore, we set the NLFM parameters to the random numbers in the above interval, and generate initial values of the sub-chirp rate PM code matrix and sub-chirp sequence code matrix using random numbers and permutations. The initial solution of the optimization $X^{\left(0\right)}$ is defined as follows.

(26) $$X^{\left(0\right)}=(X_{1}^{\left(0\right)},X_{2}^{\left(0\right)},X_{3}^{\left(0\right)})\in \Omega ,$$

### 3.2. NLFM Parameters Optimization Based on PSO

Every time after initializing the OFDM-NLFM waveform set parameters or executing of one iteration of alternating optimization, the PSO algorithm is used to optimize the NLFM parameters. According to Equation (23), the number of NLFM parameters is 2*M*. Thus the particle dimension is 2*M*. The interval of the NLFM parameters is a∈(0,0.9),b∈(−0.2,0.2). According to the global optimization model in Equation (25), the optimization model of the PSO algorithm can be expressed as

(27) $$\begin{array}{ll} \underset{X_{1}}{\min} & G\left(X_{1}\right)=ℱ\left(X_{1},X_{2}^{\left(k\right)},X_{3}^{\left(k\right)}\right)=F_{2}\left({ϒ}_{2}\left({ϒ}_{1}\left({ϒ}_{0}\left(X_{1},t_{1},t_{2},\ldots ,t_{N}\right),X_{2}^{\left(k\right)}\right),X_{3}^{\left(k\right)}\right)\right)\\ \;s.t.\; & X_{1}=\left[\begin{array}{cccc} \begin{array}{l} a_{1}\\ b_{1} \end{array} & \begin{array}{l} a_{2}\\ b_{2} \end{array} & \begin{array}{l} \cdots \;a_{m}\;\cdots \\ \cdots \;b_{m}\;\cdots \end{array} & \begin{array}{l} a_{M}\\ b_{M} \end{array} \end{array}\right]\\ & a_{m}\in \left(0,0.9\right),b_{m}\in \left(-0.2,0.2\right) \end{array},$$

where $G\left(X_{1}\right)$ represents the objective function of the PSO. $X_{2}^{\left(k\right)}$ and $X_{3}^{\left(k\right)}$ represents the current optimal sub-chirp rate PM code matrix and sub-chirp sequence code matrix after the *k*-th iteration of the alternating optimization. Note that they are the initial solution described in Equation (26) when *k =* 0. Since the PSO algorithm is executed iteratively, the initial population of the *k*+1-th iteration should include the optimal NLFM parameters in the *k*-th iteration. Thus, the population of the *k*+1-th iteration is initialized as

(28) $$P_{0}^{\left(k+1\right)}=\left\{p_{1},p_{2},p_{3},\ldots ,p_{q}\right\},$$

where $P_{0}^{\left(k+1\right)}$ is the initial population. *q* is the number of particles. $p_{1}=X_{1}^{\left(k\right)}$ is assigned to the optimal solution after the *k*-th execution of the PSO. The rest of the particles are initialized by random numbers. Initializing the population in this way ensures the current best solution to be passed to the next generation. After the *k*+1-th PSO algorithm has been executed, the optimal solution can be expressed as

(29) $$X_{1}^{\left(k+1\right)}=p_{best}\in P_{G}^{\left(k+1\right)},$$

where $P_{G}^{\left(k+1\right)}$ represents the *G*-th generation population after the *k*+1-th execution of PSO. *G* is the maximum generation of the PSO algorithm. $p_{\mathrm{best}}$ is the optimal solution, and the current optimal NLFM parameter is $X_{1}^{\left(k+1\right)}$. Recall that before each execution of the PSO algorithm, the NLFM parameters, sub-chirp rate PM code matrix, and sub-chirp sequence code matrix are set to the current optimal values to ensure that the objective function decreases monotonously.

### 3.3. Sub-Chirp Rate PM and Sub-Chirp Sequence Code Matrix Optimization Based on BCD

After obtaining current optimal NLFM parameters by the PSO, the next step of the OFDM-NLFM waveform set design algorithm is to optimize the sub-chirp rate PM code matrix and sub-chirp sequence code matrix. This task is an unconstrained combinatorial optimization problem that can be expressed as

(30) $$\begin{array}{l} \underset{X_{2},X_{3}}{\min}ℋ\left(X_{2},X_{3}\right)=ℱ\left(X_{1}^{\left(k+1\right)},X_{2},X_{3}\right)=F_{2}\left({ϒ}_{2}\left({ϒ}_{1}\left({ϒ}_{0}\left(X_{1}^{\left(k+1\right)},t_{1},t_{2},\ldots ,t_{N}\right),X_{2}\right),X_{3}\right)\right)\\ \;s.t.\;(X_{1}^{\left(k+1\right)},X_{2},X_{3})\in \Omega \end{array},$$

where $ℋ\left(X_{2},X_{3}\right)$ is the objective function of the BCD algorithm. The solution space of the optimization variables is a subspace of the solution space Ω. The goal of the above optimization is to obtain the optimal sub-chirp rate PM code matrix and sub-chirp sequence code matrix under the current optimal NLFM parameters. The optimal solutions are expressed as $X_{2}^{\left(k+1\right)}$, $X_{3}^{\left(k+1\right)}$ which satisfy

(31) $$ℱ\left(X_{1}^{\left(k+1\right)},X_{2}^{\left(k+1\right)},X_{3}^{\left(k+1\right)}\right)\leq ℱ\left(X_{1}^{\left(k+1\right)},X_{2}^{\left(k\right)},X_{3}^{\left(k\right)}\right),$$

where $X_{2}^{\left(k\right)}$ and $X_{3}^{\left(k\right)}$ are the optimal values of the sub-chirp rate PM code matrix and sub-chirp sequence code matrix after the *k*-th execution of the sub-algorithm. These two current optimal values are also the initial solutions of the *k*+1-th optimization. The optimization algorithm based on BCD proposed in this section is mainly built on the coordinate descent algorithm. The two code matrices are divided into smaller blocks. Each time we update one element or one block of the matrix, the values of the other elements are fixed. Considering that each row of the matrix corresponds to each transmit signal of the OFDM-NLFM waveform set, the above two matrices can be divided into *M* blocks according to different rows.

(32) $$\left\{ \begin{array}{l} X_{2}={\left[\begin{array}{cccc} \alpha^{1} & \alpha^{2} & \cdots & \alpha^{M} \end{array}\right]}^{T}\\ \alpha^{m}={\left[\begin{array}{cccc} \alpha_{1}^{m} & \alpha_{2}^{m} & \cdots & \alpha_{N}^{m} \end{array}\right]}^{T}\\ \alpha_{n}^{m}\in \left\{1,-1\right\},m=1,2,\ldots ,M,n=1,2,\ldots N \end{array}\right.,$$

(33) $$\left\{ \begin{array}{l} X_{3}={\left[\begin{array}{cccc} \beta^{1} & \beta^{2} & \cdots & \beta^{M} \end{array}\right]}^{T}\\ \beta^{m}={\left[\begin{array}{cccc} \beta_{1}^{m} & \beta_{2}^{m} & \cdots & \beta_{N}^{m} \end{array}\right]}^{T}\\ \;\beta_{1}^{m},\beta_{2}^{m},\ldots ,\beta_{N}^{m}\mathrm{is}\;a\;\mathrm{permutation}\;\mathrm{of}1,2,\ldots ,N,m=1,2,\ldots ,M\; \end{array}\right..$$

The blocks of the above-mentioned matrix are divided as shown in Equations (32) and (33). In the BCD algorithm, one row of the matrix is updated with the rows corresponding to the other transmit signals unchanged. Each row of the matrices is updated alternately. Therefore, the optimization algorithm proposed in this paper is composed of the two layers of loops:

**Outer loop:** Update the *m*-th row vector $\alpha^{m}$ of $X_{2}^{\left(k\right)}$ and $\beta^{m}$ of $X_{3}^{\left(k\right)}$. The values of the other row vectors remain unchanged. The 1, 2,…, *M*-th row of the matrices are updated alternately until the objective function converges.

**Inner loop:** Update the *n*-th column of the row vector $\alpha^{m}$ and $\beta^{m}$, keeping the other columns unchanged. The 1, 2,…, *N*-th columns are updated alternately.

Therefore, the original high-dimensional optimization problem shown in Equation (30) is equal to solving several low-dimensional optimization problems. The optimization variable after the *i*-th outer loop is

(34) $$\begin{array}{l} X_{2,\;(i)}={\left[\begin{array}{cccc} \alpha_{\left(i\right)}^{1} & \alpha_{\left(i\right)}^{2} & \cdots & \alpha_{\left(i\right)}^{M} \end{array}\right]}^{T}\\ X_{3,\;(i)}={\left[\begin{array}{cccc} \beta_{\left(i\right)}^{1} & \beta_{\left(i\right)}^{2} & \cdots & \beta_{\left(i\right)}^{M} \end{array}\right]}^{T} \end{array},$$

where $X_{2,\;(0)}=X_{2}^{\left(k\right)}$ and $X_{3,\;(0)}=X_{3}^{\left(k\right)}$. The outer loop execution can also be summarized as a minimization problem as follows.

(35) $$\begin{array}{l} \underset{\alpha^{m},\beta^{m}}{\min}ℋ\left(\alpha^{m},\beta^{m},\left[\alpha_{\left(i\right)}^{1},\alpha_{\left(i\right)}^{2},\ldots ,\alpha_{\left(i\right)}^{m-1},\alpha_{\left(i\right)}^{m+1},\ldots ,\alpha_{\left(i\right)}^{M}\right],\left[\beta_{\left(i\right)}^{1},\beta_{\left(i\right)}^{2},\ldots ,\beta_{\left(i\right)}^{m-1},\beta_{\left(i\right)}^{m+1},\ldots ,\beta_{\left(i\right)}^{M}\right]\right)\\ \;s.t.\;\alpha^{m}\in {\left\{1,-1\right\}}^{N},\beta^{m}\in G_{N} \end{array},$$

where ${\left\{1,-1\right\}}^{N}$ is the set of the binary row vectors whose length are *N*. *G_N_* is the set of all possible permutations of 1, 2, …, *N*. The number of the element of the set is *N*! and the optimization solution is expressed as

(36) $$\begin{array}{l} X_{2,\;(i+1)}^{*}=\left[\alpha_{\left(i\right)}^{1},\alpha_{\left(i\right)}^{2},\ldots ,\alpha_{\left(i\right)}^{m-1},\alpha_{*}^{m},\alpha_{\left(i\right)}^{m+1},\ldots ,\alpha_{\left(i\right)}^{M}\right]\\ X_{3,\;(i+1)}^{*}=\left[\beta_{\left(i\right)}^{1},\beta_{\left(i\right)}^{2},\ldots ,\beta_{\left(i\right)}^{m-1},\beta_{*}^{m},\beta_{\left(i\right)}^{m+1},\ldots ,\beta_{\left(i\right)}^{M}\right] \end{array},$$

where $\alpha_{*}^{m}$ and $\beta_{*}^{m}$ are the optimal row vectors, $X_{2,\;(i+1)}^{*}$ and $X_{3,\;(i+1)}^{*}$ is the optimal code matrices of the *i*+1-th iteration. In the next iteration, we change the value of *m* and optimize another row of the sub-chirp rate PM code matrix and sub-chirp sequence code matrix. The process of the outer loop can be summarized in Algorithm 1.
**Algorithm 1:** Block coordinate descent algorithm for *X*_2_ and *X*_3_**Input:** Initial solution $X_{2}^{\left(k\right)},X_{3}^{\left(k\right)}$.**Output:** Optimal solution $X_{2}^{\left(k+1\right)},X_{3}^{\left(k+1\right)}$.**Step 1:** *m* = 1, *i* = 0, $X_{2,\;(0)}=X_{2}^{\left(k\right)}$, $X_{3,\;(0)}=X_{3}^{\left(k\right)}$.**Step 2:** Update the *m*-th row vector $\alpha_{\left(i\right)}^{m}$ of $X_{2,\;(i)}$ and $\beta_{\left(i\right)}^{m}$ of $X_{3,\;(i)}$. Input it to **Algorithm 2** whose output is the optimal row vector $\alpha_{*}^{m}$ and $\beta_{*}^{m}$.**Step 3:**$\left\{ \begin{array}{l} X_{2,\;(i+1)}=\left[\alpha_{\left(i\right)}^{1},\alpha_{\left(i\right)}^{2},\ldots ,\alpha_{\left(i\right)}^{m-1},\alpha_{*}^{m},\alpha_{\left(i\right)}^{m+1},\ldots ,\alpha_{\left(i\right)}^{M}\right]\\ X_{3,\;(i+1)}=\left[\beta_{\left(i\right)}^{1},\beta_{\left(i\right)}^{2},\ldots ,\beta_{\left(i\right)}^{m-1},\beta_{*}^{m},\beta_{\left(i\right)}^{m+1},\ldots ,\beta_{\left(i\right)}^{M}\right] \end{array}\right.$**Step 4:** *i* = *i* + 1. If m>M is true, *m* = 1, otherwise *m* = *m* + 1.**Step 5:** Calculate the objective function using $X_{2,\;(i)}$ and $X_{3,\;(i)}$. If the value of the objective function is not decreasing, $X_{2}^{\left(k+1\right)}=X_{2,\;(i)},X_{3}^{\left(k+1\right)}=X_{3,\;(i)}$ and the optimal matrix is obtained, otherwise jump to Step 2 and continue.

The inner loop calculates the optimal value of $\alpha_{*}^{m}$, $\beta_{*}^{m}$. Similarly, denote *j* be iteration counter, and the row vector after the *j*+1-th iteration can be expressed as

(37) $$\begin{array}{l} \alpha^{m,\;(j+1)}=\left[\begin{array}{cccc} \alpha_{1}^{m,\;(j)} & \alpha_{2}^{m,\;(j)} & \cdots & \alpha_{N}^{m,\;(j)} \end{array}\right]\\ \beta^{m,\;(j+1)}=\left[\begin{array}{cccc} \beta_{1}^{m,\;(j)} & \beta_{2}^{m,\;(j)} & \cdots & \beta_{N}^{m,\;(j)} \end{array}\right] \end{array}.$$

When *j* continues to increase until the value of the objective function converges, and the obtained optimal solution is $\alpha_{*}^{m}$, $\beta_{*}^{m}$. In order to describe the update of the time-frequency code matrix, consisting of *M* permutations, the exchange operation of the row vector or the permutation is defined as follows

(38) $$\beta^{m,\;(j+1)}•\left(N_{b},N_{a}\right)=\left[\beta_{1}^{m,\;(j)},\ldots ,\beta_{b}^{m,\;(j)},\ldots ,\beta_{a}^{m,\;(j)},\ldots ,\beta_{N}^{m,\;(j)}\right],$$

where $•\left(N_{a},N_{b}\right)$ represents the exchange of the *a*-th and *b*-th column of the row vector. According to the definition of the exchange operator, the update algorithm of the row vector can be summarized as in Algorithm 2.
**Algorithm 2:** The update algorithm for the *m*-th row of *X*_2_ and *X*_3_**Input:** Current optimal row vectors $\alpha_{\left(i\right)}^{m}$, $\beta_{\left(i\right)}^{m}$ from **Algorithm 1**.**Output:** Optimal row vectors $\alpha_{*}^{m}$, $\beta_{*}^{m}$.**Step 1:** *n* = 1, *j* = 0, $\beta^{m,\;(0)}=\beta_{\left(i\right)}^{m}$, $\alpha^{m,\;(0)}=\alpha_{\left(i\right)}^{m}$.**Step 2:** Exchange every columns with the *n*-th column of $\beta^{m,\;(j)}$, and *N* new row vectors is obtained as $\beta^{m,\;(j)}•\left(N_{1},N_{n}\right),\ldots ,\beta^{m,\;(j)}•\left(N_{N},N_{n}\right)$.**Step 3:** Calculate *N* objective function ${ℱ}_{1},{ℱ}_{2},\ldots ,{ℱ}_{N}$ using *N* new row vectors. Select the minimum objective function ${ℱ}_{l}$ (if the *l*-th value is lowest) and its corresponding row vector is optimal solution expressed as $\beta^{m,\;(j+1)}=\beta^{m,\;(j)}•\left(N_{l},N_{n}\right)$.**Step 4:** The value of the *n*-th column of $\alpha^{m,\;(j)}$ satisfies $\alpha_{n}^{m,\;(j+1)}\in \left\{1,-1\right\}$. Select the one making the objective function is the lowest and the optimal solution is $\alpha^{m,\;(j+1)}$.**Step 5:***j* = *j* + 1. If n>N is true, *n* = 1, otherwise *n* = *n* + 1.**Step 6:** Calculate the objective function using $\alpha^{m,\;(j)}$, $\beta^{m,\;(j)}$. If the value of the objective function is not decreasing, the optimal row vectors $\alpha_{*}^{m}$, $\beta_{*}^{m}$ is obtained, otherwise jump to Step 2 and continue.

## 4. Numerical Simulation

### 4.1. OFDM-NLFM Waveform Set Design

This section designs the OFDM-NLFM waveform set using the proposed algorithm based on alternating optimization in Section 3. We evaluate the performance indicators PASR and PCCR in Equations (21) and (22) under typical radar system parameters. The result is compared with the IN-OFDM that currently has the best correlation function performance. The system parameters in this section are shown in Table 1. The numerical simulations of the proposed OFDM-NLFM waveform set design algorithm are implemented with MATLAB that runs on a PC with one Intel Core i7-6700 CPU and 8 GB RAM. Numerical simulations in the subsequent sections are also implemented in the same way. To compare with IN-OFDM, the time-domain segmentation parameters in Equation (13) are set as $t_{1}^{m},t_{2}^{m},\ldots ,t_{8}^{m}=0.08,0.40,0.72,2.80,2.80,0.72,0.4,0.08\;\mu s$. The segmentation parameters of different transmit signals are the same when *m* = 1, 2, 3, 4.

**Figure 6:**
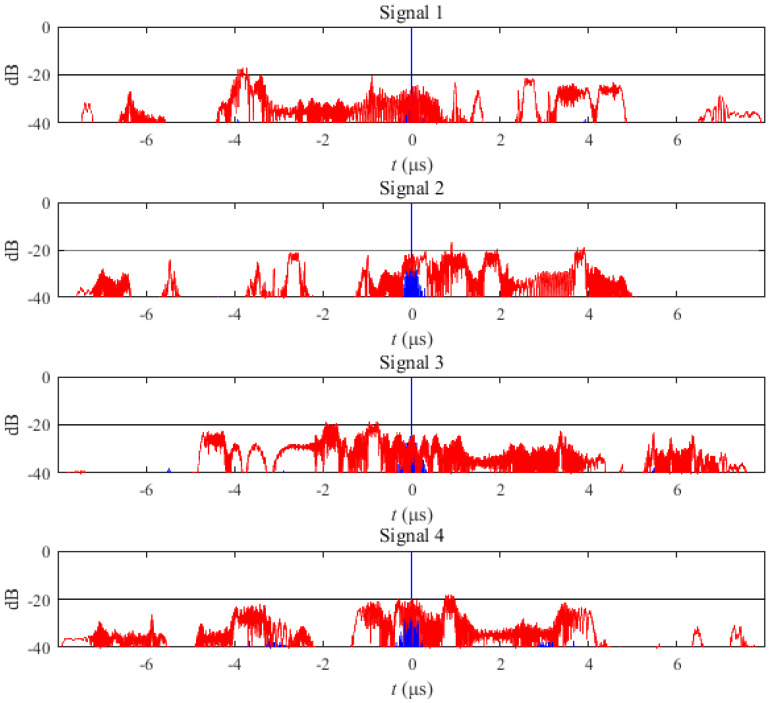
Auto-correlation function and cross-correlation function of the transmit signals of the IN-OFDM waveform set.

The correlation function of the IN-OFDM waveform is shown in Figure 6. The blue curve in Figure 6 represents the auto-correlation function of the IN-OFDM waveform set for each transmit signal, and the red curve is the cross-correlation function between the sum of the other transmit signals and itself. Results show that PASR = −17.7259 dB, PCCR = −16.4961 dB. The specific values of the sub-chirp rate PM code matrix and sub-chirp sequence code matrix are shown in Table I of [41].

**Figure 7:**
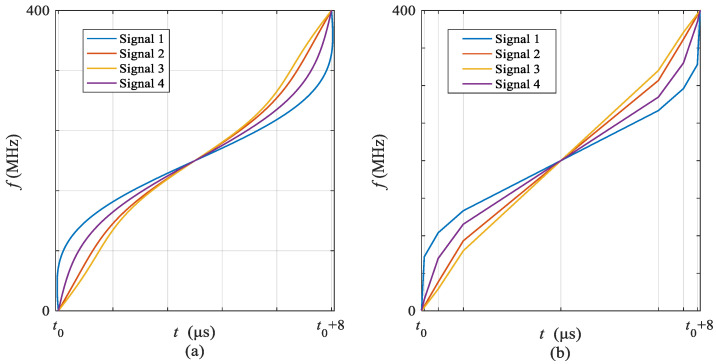
Time-frequency structure (**a**) NLFM signals, (**b**) piecewise chirp signals.

**Figure 8:**
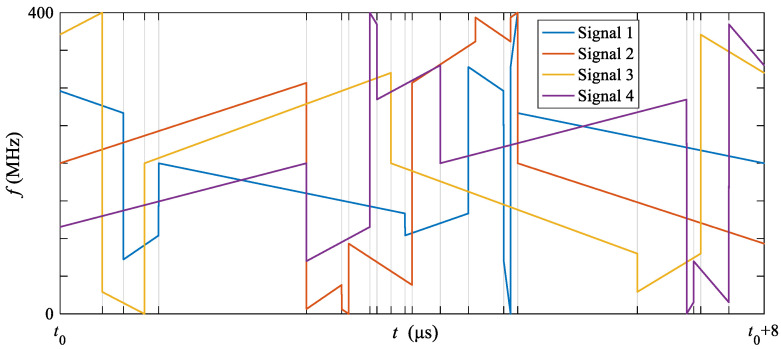
Time-frequency structure of the transmit signals of the OFDM-NLFM waveform set obtained by the proposed algorithm.

**Figure 9:**
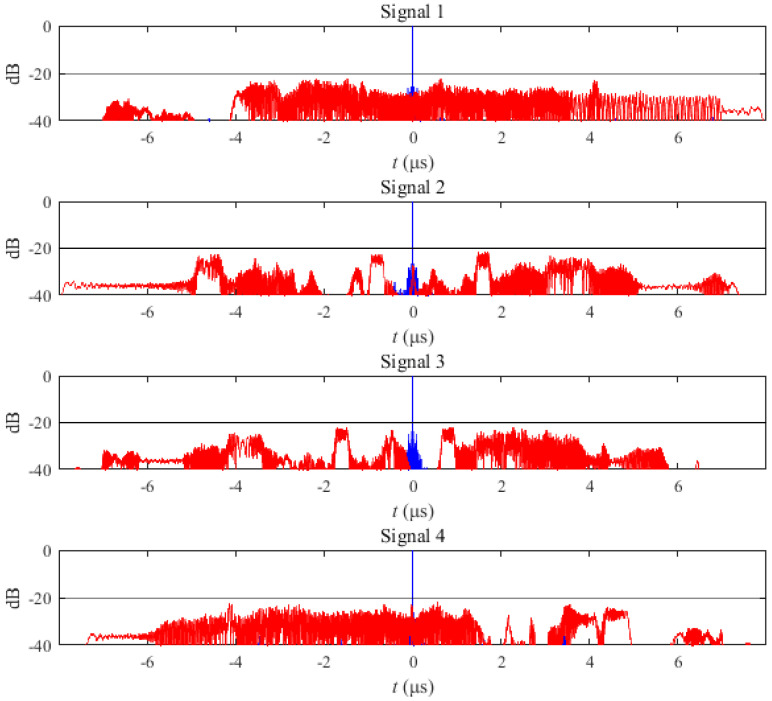
Auto-correlation function and cross-correlation function of the transmit signals of the OFDM-NLFM waveform set obtained by the proposed algorithm.

Under the same setting, the sub-chirp rate PM code matrix and sub-chirp sequence code matrix obtained by the proposed OFDM-NLFM waveform set design algorithm are shown in Table 2 and Table 3. The indicator values of the optimized OFDM-NLFM waveform set are PASR = −21.7567 dB, PCCR = −21.7806 dB. Its PASR is 4.03 dB lower than that of the IN-OFDM waveform set. Its PCCR is 5.2845 dB lower than that of the IN-OFDM waveform. Figure 7 is the obtained time-frequency structure of the NLFM signals and the piecewise chirp signals. Figure 8 is the time-frequency structure of the transmit signals of the OFDM-NLFM waveform set obtained by the proposed algorithm. The blue curve in Figure 9 represents the auto-correlation function of the OFDM-NLFM waveform set for each transmit signal, and the red curve is the cross-correlation function between the sum of the other transmit signals and itself.

### 4.2. The Effect of Random Initialization

This section analyzes the influence of different random initial values on the final design result. This section randomly generates 10 groups of sub-chirp rate PM code matrix and sub-chirp sequence code matrix as the initial solution for the proposed optimization algorithm. We calculate the MIMO waveform set correlation function indicator under the same radar system parameters in Table 1. The time-domain segmentation parameters are still set to $t_{1}^{m},t_{2}^{m},\ldots ,t_{8}^{m}=0.08,0.40,0.72,2.80,2.80,0.72,0.4,0.08\;\mu s$. The results are shown in Table 4. 

**Figure 10:**
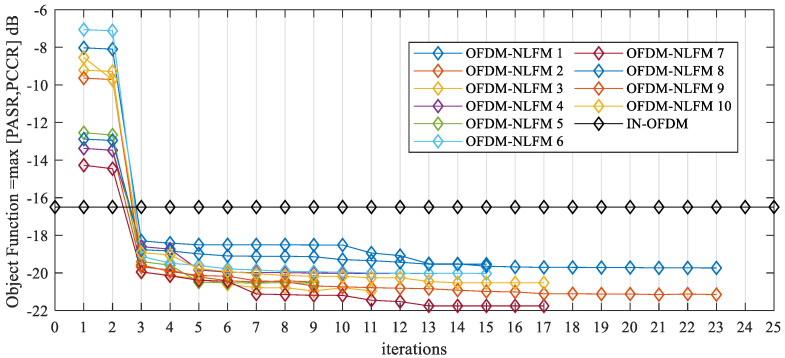
Convergence curve of objective function of OFDM-NLFM waveform set when randomly initialized.

It can be seen from Table 4 that the average value of the PASR is −20.4754 dB and the standard deviation is 0.6808 dB. The average value of the PCCR is −20.6389 dB, and the standard deviation is 0.6885 dB. They are much lower than that of IN-OFDM. The standard deviation of the 10 sets of data does not exceed 0.7 dB, which means that the result of the proposed method is stable. The convergence curves of the objective function are shown in Figure 10. The horizontal axis is the number of iterations. When the number of iterations is odd, the objective function values are obtained by the PSO algorithm. When the number of iterations is even, the objective function values are obtained by the BCD algorithm. The objective function is max [PASR, PCCR] as in Equation (24). According to the results of 10 experiments, the initial objective function value ranges from −7.06 dB to −14.27 dB. After the proposed algorithm has been executed, the objective function value decreases to values around −20.00 dB. The results show that the proposed algorithm based on alternating optimization can obtain the MIMO radar OFDM-NLFM waveform set with low PASR and PCCR.

### 4.3. The Effect of Time-Domain Segmentation Parameters

Here we keep the radar system parameters unchanged, as shown in Table 1. This section analyzes the influence of time-domain segmentation parameters on the results of the OFDM-NLFM waveform set. This section changes the time-domain segmentation parameters to a uniform division as $t_{n}^{m}=1.00\;\mu s,n=1,2,\ldots ,8$. The results are obtained after initializing 10 sets of random solutions. The PASR and PCCR values of the obtained waveform set are shown in Table 5. It can be seen from the results that the average value of the PASR obtained by the alternating optimization design is −19.0154 dB, with a standard deviation being 1.0923 dB. The average value of PCCR is −19.0118 dB, and the standard deviation is 1.0925 dB. Except for the time-domain segmentation parameters, other settings of the above 10 tests are the same as in Table 4. It can be seen that the average value of PASR and PCCR in Table 5 is higher than that in Table 4, and the standard deviation is higher in Table 5. Therefore, the time-domain segmentation parameters should be appropriately selected when using the proposed algorithm to design the OFDM-NLFM waveform set.

### 4.4. The Effect of MIMO System Parameters

**Figure 11:**
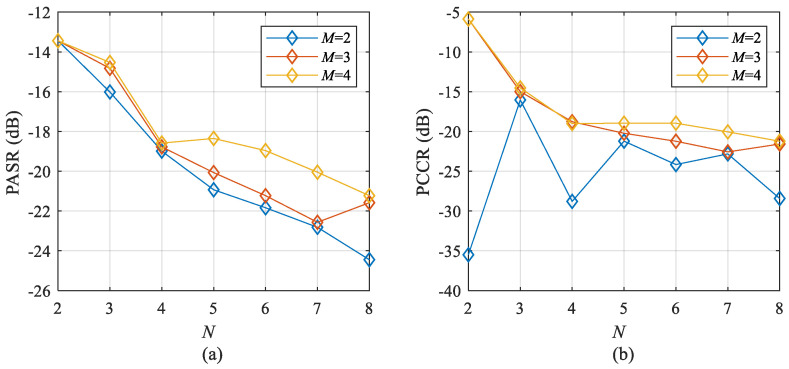
The PASR and PCCR of the waveform obtained by the propoesd algorithm under different *M* and *N* (**a**) PASR, (**b**) PCCR.

In order to analyze the result of the proposed OFDM-NLFM waveform set design algorithm based on alternating optimization under different MIMO system parameters, this section changes the number of transmit signals *M* and the number of sub-bands *N,* respectively. The bandwidth, pulse width, and sampling frequency are still the same as in Table 1. Set the number of transmit signals *M* to {2,3,4}, and the number of sub-bands *N* to {2,3,4,5,6,7,8}. The time-frequency segmentation parameters are set to a uniform segmentation. The indicator values of the waveform set obtained by the proposed algorithm are shown in Figure 11. Figure 11a shows the curve of the PASR with different *M* and *N*, and Figure 11b shows the curve of PCCR with different *M* and *N*. From the results in the previous section, it is known that the proposed algorithm is less affected by the initialization. The following results are obtained by a single test. It can be seen that the larger the *M*, the larger the PASR and PCCR, which match the common facts. However, when *M* = 2, *N* = 2, PCCR is the lowest. No matter how large the *N* is, it seems that there cannot be a lower cross-correlation function peak. The reason is that the cross-correlation function between an up-chirp signal and a down-chirp signal is the lowest. When *M* > 2, the larger the *N* is, the lower the peak value of the cross-correlation function. Furthermore, the PASR and PCCR will increase rapidly as *M* increase. The design freedoms of the OFDM-NLFM waveform set are low when *M* is larger than 4.

## 5. Conclusions

This paper constructs a signal model of the MIMO radar OFDM-NLFM waveform set, and the task is modeled as a complex NP-hard nonlinear and non-convex optimization problem. To solve this optimization problem, we propose an OFDM-NLFM waveform set design algorithm based on alternating optimization. The proposed algorithm uses the PSO algorithm to optimize the continuous NLFM parameters and uses the BCD algorithm to optimize the discrete sub-chirp rate PM code matrix and sub-chirp sequence code matrix. The two sub-steps are executed alternately until the objective function converges. The numerical simulation results show that the OFDM-NLFM waveform set designed by the proposed optimization algorithm is better than that of currently the best IN-OFDM waveform set. The value of PASR is 4.03 dB lower than that of the IN-OFDM waveform set. The PCCR is 5.2845 dB lower than that of the IN-OFDM waveform set. In addition, we also compare the obtained waveform set under different time-domain segmentation parameters and MIMO radar system parameters. Results show that the time-domain segmentation parameters have an influence on the results, and therefore a proper selection is required.

## Figures and Tables

**Table 1:** MIMO radar system parameter.

Parameters	Values
Pulse Width (*T*)	8 μs
Sample frequency (Fs)	800 MHz
Bandwidth (B)	400 MHz
Signal Number (*M*)	4
Sub-band Number (*N*)	8

**Table 2:** Sub-chirp rate PM code matrix of the optimal OFDM-NLFM waveform set.

*X*_2_	Chirp 1	Chirp 2	Chirp 3	Chirp 4	Chirp 5	Chirp 6	Chirp 7	Chirp 8
Signal 1	−1	1	−1	1	−1	−1	1	−1
Signal 2	1	1	−1	−1	1	−1	1	−1
Signal 3	1	1	−1	−1	1	−1	1	−1
Signal 4	1	1	−1	1	1	1	−1	−1

**Table 3:** Sub-chirp sequence code matrix of the optimal OFDM-NLFM waveform set.

*X*_3_	Chirp 1	Chirp 2	Chirp 3	Chirp 4	Chirp 5	Chirp 6	Chirp 7	Chirp 8
Signal 1	6	2	4	3	7	1	8	5
Signal 2	5	2	1	3	6	7	8	4
Signal 3	7	8	2	1	5	4	3	6
Signal 4	4	3	8	6	5	1	2	7

**Table 4:** OFDM-NLFM waveform set design results when the segmentation parameters set to $t_{1}^{m},t_{2}^{m},\ldots ,t_{8}^{m}=0.08,0.40,0.72,2.80,2.80,0.72,0.4,0.08\;\mu s$ .

Number	1	2	3	4	5
PASR	−19.7342	−20.4841	−20.9608	−20.032	−20.5363
PCCR	−19.7343	−20.4871	−21.0856	−20.0368	−20.7768
	**6**	**7**	**8**	**9**	**10**
PASR	−20.0301	−21.7567	−19.536	−21.1542	−20.5295
PCCR	−20.3785	−21.7806	−19.7171	−21.2282	−21.1641

**Table 5:** OFDM-NLFM waveform set design results when the segmentation parameters set to $t_{n}^{m}=1.00\;\mu s,n=1,2,\ldots ,8$ .

Number	1	2	3	4	5
PASR	−18.1208	−19.7324	−17.9071	−19.7083	−18.9741
PCCR	−18.1192	−19.733	−17.9083	−19.7083	−18.937
	**6**	**7**	**8**	**9**	**10**
PASR	−18.1787	−19.8441	−18.5468	−21.2277	−17.9141
PCCR	−18.1789	−19.8441	−18.5468	−21.2279	−17.9149

## Data Availability

Not applicable.
